# QTL Mapping and Marker Identification for Sex-Determining: Indicating XY Sex Determination System in the Swimming Crab (*Portunus trituberculatus*)

**DOI:** 10.3389/fgene.2018.00337

**Published:** 2018-08-23

**Authors:** Jianjian Lv, Dongfang Sun, Pengpeng Huan, Liu Song, Ping Liu, Jian Li

**Affiliations:** ^1^Key Laboratory of Sustainable Development of Marine Fisheries, Ministry of Agriculture, Yellow Sea Fisheries Research Institute, Chinese Academy of Fishery Sciences, Qingdao, China; ^2^Laboratory for Marine Fisheries and Aquaculture, Qingdao National Laboratory for Marine Science and Technology, Qingdao, China

**Keywords:** QTL, sex marker, XY sex determination system, *Portunus trituberculatus*, sex-related gene

## Abstract

Sex determination is an important area of research, which has always had an intriguing aspect in evolutionary and developmental biology. Quantitative trait locus (QTL) mapping for sex will be helpful in clarifying the sex determination system. In this study, the sex QTL mapping of the swimming crab (*Portunus trituberculatus*) was performed based on a high-density linkage map, and a highly significant QTL specifically mapped on a single linkage group (LG) was firstly identified (LG24, LOD > 14). Twenty markers in the QTL region showed significant associations with sex by association analysis, of which heterogametic genotypes in males supported the XY sex determination mechanism. Two sex-specific markers at the family level were identified via segregation distortion analysis, which were known to be the most closely linked to the sex of *P. trituberculatus*. Based on sex marker sequences (Marker3840, Marker20320, and Marker10494), three potential sex-related genes were identified, and the quantitative real-time PCR results suggested that these genes were important in spermatogenesis or sex characteristics in males. Our results will contribute to the fine-mapping of sex-determining genes and clarify the sex determination mechanism of *P. trituberculatus*.

## Introduction

The swimming crab (*Portunus trituberculatus*) is distributed widely along the coastal waters of China, Korea, Japan, and other East Asian countries (Lv et al., 2013), and has become one of the most important economic species with an annual production of 605,632 tons (Ren and Pan, 2014; Food and Agriculture Organization [FAO], 2016; Meng et al., 2018). In China, the swimming crab is a dominant crustacean species in mariculture, the total production reached 125,317 tons in 2016 (Fishery Bureau, Ministry of Agriculture of the People’s Republic of China, 2016).

Sex determination is known as a plastic biological developmental process, which has always had an intriguing aspect in evolutionary and developmental biology (Charlesworth and Mank, 2010). The genetic mechanism of sex determination of vertebrates commonly shows male heterogamety (XY) or female heterogamety (ZW) mechanism (Koopman, 2001). In crustaceans animals, the mechanisms of sex determination are remarkably diverse and are controlled by genetic and/or environmental factor (Ford, 2008). The sex determination system of some crustaceans can be identified by karyotype analysis (Abu-Almaaty, 2017; Blackmon et al., 2017). In crabs, four species (*Eriocheir japonica*, *Hemigrapsus sanguineus*, *Hemigrapsus penicillatus*, and *Plagusia dentipes*) were believed to have an XY sex determination system via karyotype analysis (Niiyama, 1937, 1938, 1959; Lecher and Noel, 1995). However, due to a large numbers of chromosomes and complex genomes, sex determination by karyotype analysis cannot be determined in most crustaceans (Torrecilla et al., 2017). In addition, a recent study questioned the reliability of karyotype analysis for inferring sex determination, as the centromeres could not be identified in some of the chromosomes (Lee et al., 2004).

Recent genetic studies based on high- density linkage maps have provided new insights into sex determination systems and genome organization of sex chromosomes in crustaceans animals. The linkage-mapping analysis of *E. sinensis, Litopenaeus vannamei*, *Penaeus monodon*, and *P. japonicus* detected major sex-determining quantitative trait loci (QTLs) in one LG (Li et al., 2003; Pérez-Rostro and Ibarra, 2003; Perez et al., 2004; Zhang et al., 2007; Du et al., 2010; Robinson et al., 2014; Cui et al., 2015; Alcivarwarren et al., 2017; Qiu et al., 2017), which indicated the existence of a sex chromosome. In addition, the genotypes of markers located on the potential sex chromosome could also be used to judge the type of sex determination system. In *E. sinensis*, all sex-associated markers were located on a single linkage group (LG60). In addition, 46 markers detected by genome-wide association studies (GWASs) were heterozygous and segregated only in the female parent, which documents a ZW sex determination system in *E. sinensis* (Cui et al., 2015). In *L. vannamei*, the sex determination region was fine-mapped to a small region along LG18 via the integration of linkage and association analysis (Yang et al., 2017). One fully sex-associated marker was identified, supporting the ZW sex determination mechanism in *L. vannamei*. However, although clarifying the mechanism of sex determination has great implications in *P. trituberculatus*, as female crabs are a higher economic value than male, the studies of QTL mapping and marker identification for sex have not yet been reported.

Recently, a high-density linkage map with 10,963 markers mapped to 53 sex-averaged LGs, has been constructed for *P. trituberculatus* in our group (Lv et al., 2017). The average marker distance was 0.51 cM, which provided a solid foundation for the fine QTL mapping of sex. The aim of the present study was to identify the QTL and markers for sex determination in *P. trituberculatus*. QTL mapping was performed based on the high-density linkage map constructed previously. Association and segregation distortion (SD) analysis was carried out to further identify sex markers. As a result, the sex QTL was specifically mapped to LG24 and two sex markers with a complete association to sex at the family level were identified. The results supported the male heterogametic (XY) sex determination mechanism in *P. trituberculatus* and will contribute to the fine-mapping of sex-determining genes with the help of genome sequencing in the future.

## Materials and Methods

### Ethics Statement

This study was carried out in accordance with the recommendations of ‘the Yellow Sea Fisheries Research Institute.’ The protocol was approved by ‘the Yellow Sea Fisheries Research Institute.’ The crabs used in the present study were marine-cultured animals, and all of the experiments were conducted according to the regulations of the local and central government. Prior to the sampling, all crabs were treated with cold shock method to minimize suffering.

### Experimental Crab and Data Collection

The QTL mapping population was same as the mapping population in our previous study (Lv et al., 2017), which was created by artificial insemination using a male parent from a F9 full sibling and a female parent from the wild population of the Bohai Sea, China. Briefly, a total of 116 progeny were randomly selected at harvest time. Genomic DNA were extracted using TIANamp Marine animal DNA extraction kit (Catalog Number: DP324, TIANGEN, Beijing, China). A linkage map with 10,963 markers was constructed (marker interval 0.51 cM) via SLAF-seq (specific-locus amplified fragment sequencing) (Lv et al., 2017). Sequencing was performed on the Illumina HiSeq 2500 sequencing platform (Illumina, Inc., San Diego, CA, United States) with a 150 bp read length. Sequencing raw data have been submitted to the Sequence Read Archive (SRA) database of NCBI with the accession code SUB4354630.

### QTL Mapping for Sex

Phenotypic sex was judged by observing the shape of the crab’s abdomen (Song, 1982), which was treated as a binary trait (0 for females and 1 for males). The interval mapping and multiple-QTL model mapping (MQM) were used in QTL analysis via MapQTL 4.0 software as described (Ooijen et al., 2000; Wei et al., 2014). Two-LOD support intervals were constructed as 95% confidence intervals (Ooijen, 1992). Likelihood-ratio statistic (LOD) with a minimum score of 14.0 was used to declare the significant QTL, which was determined using 1,000 permutations. The phenotypic variance explained (*PVE*) was also calculated in MapQTL4.0 based on the population variance.

### Association Analysis

As a complementary approach to QTL mapping, the relationship between markers in the linkage map and the sex phenotypic variations was further tested by association analysis in the QTL mapping population using an R package called ‘GWAF’ (Chen and Yang, 2010), which was designed mainly to analyze a batch of genotyped markers against a continuous or dichotomous phenotype measured on subjects of families for genetic association. Logistic regressions via generalized estimating equations (GEE) model and a linear mixed-effects model was used to test the genetic association for each marker. Bonferroni correction was applied to multiple comparisons for counteracting errors.

### Segregation Distortion Analysis

Quantitative trait locus mapping population was divided into female (81 individuals) and male (35 individuals) groups. Segregation distortion was examined within these female and male groups respectively using a χ^2^ goodness of fit test compared to the expected segregation ratio. Bonferroni correction was also applied to multiple comparisons for counteracting errors. The marker of significant deviation from the expected segregation ratio in the two groups is a potential sex marker (*p* < 0.05).

### Validation of Sex Markers

The potential sex markers were validated in randomly selected 30 females and 30 males of different populations by Sanger sequencing. Firstly, these markers were verified in the QTL mapping family (1^#^ full sibling family), and then were validated in another full sibling family (2^#^ full sibling family) and a wild population. Primers were designed on flanking regions in order to amplify the SNPs (**Supplementary Table S1**). The PCR amplification was performed under the following conditions: 95°C for 1 min, followed by 35 cycles of 95°C for 30 s, 55–60°C for 30 s, and 72°C for 30 s; and 72°C for 10 min. The PCR products were sequenced in both directions using ABI 3730 (Applied Biosystems, United States) with the forward and reverse primer. The genotypes were identified by checking the Sanger sequence chromatogram at the SNP position. Sequencing chromatograms were visually analyzed using the Vector NTI Suite 11.0 (Invitrogen).

### Sex-Related Genes

To detect the sex-related genes, we firstly compared our marker sequences with all available genomic scaffolds/contigs via BLAT tool (Kent, 2002). Then, compared the scaffolds/contigs sequences with the transcriptomic sequences. Thirdly, to check whether these transcriptomic sequences could be explicitly annotated via blastx with public databases. Finally, it is further screened according to the method of candidate genes by further consulting published literatures. The genomic scaffolds/contigs and transcriptomic sequences were come from our previous work (SUB2680377) (Lv et al., 2017).

### qPCR Analysis

For the genes with sex-associated markers, gene expression was analyzed at the different developmental stages of gonadal, embryos,and larvae. Gonadal tissue were from our previous collected sample containing five stages testicular sample and six stages ovariansample (Meng et al., 2018). In addition, samples of 12 different developmental stages from fertilized eggs to young crabs were also collected based on the external features and histological configuration (Yingmin et al., 1984; Jun-Zeng et al., 2001a,b), including fertilized egg stage (F), multicellular stage (Mc), blastula stage (B), gastrulation stage (G), egg-nauplius stage (En), egg-zoea stage (Ez), zoea stage (Z1–Z4), megalopal stage (M), and juvenile crab stage (J). Total RNA was extracted individually using Trizol (Invitrogen, Carlsbad, CA, United States). Quantitative real-time PCR (qPCR) primers were designed via Primer Premier 5 tool (Premier Biosoft International) (**Supplementary Table S1**). First strand cDNA was synthesized using PrimeScript RT reagent kit (Takara, Dalian, China). qPCR was performed using a 7500 Fast Real-Time PCR System and SYBRPremix^®^ Ex Taq^TM^ reagent Kit (Takara, Dalian, China). The PCR was performed with the following profile: 95°C for 30 s, followed by 40 cycles of 95°C for 15 s and 60°C for 34 s.

## Results

### QTL Mapping and Association Analysis

A highly significant QTL for sex (LOD > 14 at the whole genomic level) was mapped on integrated LG24 with a confidence interval of 43.721 cM (0.87–44.591 cM). The highest LOD score was 99.99, which was observed from 0.87 to 4.348 cM in LG24. Eighty-one markers were located in the QTL intervals, and contributed to a *PVE* of 14.15–100.00. These markers included 23 markers that segregated in the female parent, 19 segregated in the male and 39 markers were heterozygous in both parents (**Supplementary Table S2**).

**FIGURE 1:**
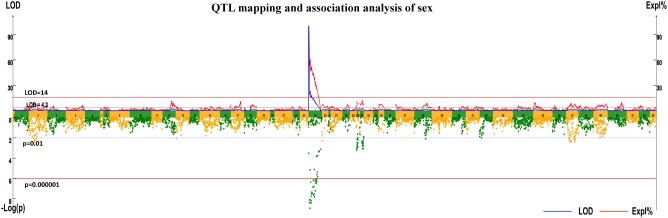
Quantitative trait locus (QTL) mapping and association analysis of sex in Portunus Trituberculatus.

Association analysis revealed 20 markers showed significant associations with sex in the mapping family, and all of them located within a region from 9.431 to 44.591 cM of LG24, which is, therefore, the putative Y chromosome (**Figure 1** and **Table 1**). Apart from Marker46734 (heterozygous in both parents), 19 markers segregated in the male (genotyped as lmxll), suggested that the sex determination of the crab may belong to the male heterogametic XY system.

**Table 1:** Candidate sex markers identified by QTL mapping and association analysis.

Number	Group	Position	Marker	LOD	%Expl	Type
1	24	9.431	Marker46391	19.03	53.2	<lmxll>
2	24	10.301	Marker6733	17.8	50.7	<lmxll>
3	24	12.055	Marker41453	20.48	55.8	<lmxll>
4	24	13.81	Marker25749	20.42	55.5	<lmxll>
5	24	17.343	Marker3097	15.8	46.6	<lmxll>
6	24	20.031	Marker37410	15.05	45.1	<lmxll>
7	24	22.253	Marker38595	16.81	48.7	<lmxll>
8	24	23.122	Marker42414	18.07	51.2	<lmxll>
9	24	23.122	Marker31025	18.07	51.2	<lmxll>
10	24	23.992	Marker54480	17.81	50.7	<lmxll>
11	24	27.501	Marker28318	15.8	46.6	<lmxll>
12	24	27.501	Marker46734	15.8	46.6	<hkxhk>
13	24	28.097	Marker43997	17.65	50.6	<lmxll>
14	24	28.372	Marker7940	17.8	50.7	<lmxll>
15	24	28.807	Marker81606	16.74	48.5	<lmxll>
16	24	31.866	Marker36368	15.22	45.3	<lmxll>
17	24	43.279	Marker42426	15.12	45.1	<lmxll>
18	24	43.279	Marker54381	15.12	45.1	<lmxll>
19	24	43.279	Marker22016	15.12	45.1	<lmxll>
20	24	44.591	Marker35698	14.21	43.1	<lmxll>

To verify the sex-associated markers, 11/20 markers with sufficient flanking regions were selected to be validated in the parent of the mapping family by sequencing the PCR product. Two markers were poorly sequenced (Marker42426 and Marker7940), however, the sequencing results of the other nine markers were all consistent with typing information of SLAF. Two of the nine markers (Marker46391 and Marker3097) were further selected to be validated in the mapping population and another full sibling family, and both of them were significantly associated with the sex traits in the two families (*p* < 0.01). The results suggest that screening sex-associated markers through QTL and association analysis was reliable. However, according to the marker genotyping results via SLAF analysis (**Supplementary Table S3**), none of the 20 markers showed complete association with sex.

### Sex Markers Identification

As no marker identified by linkage mapping was perfectly associated with sex in the validated populations, we applied a SD analysis approach to further screen the sex markers in addition to the markers on the genetic linkage map. The results showed that 416 markers significantly deviated from the expected segregation ratio in both male and female groups (522 SD markers in male group and 1,022 in the female group) (**Supplementary Tables S4**, **S5**). Filtering out the markers of distored in the same direction in both sexes (e.g., there is an excess of heterozygotes in both males and females), we found 179 markers distored in opposite directions between the sexes (**Supplementary Table S6**). Interestingly, all of these makers appear to be segregating in the father, which was another strong evidence of XY sex determination system. Seventeen markers highly significant deviated from the expected segregation ratio were further screened out based on the *p*-value (*p* = 0 in female and *p* < 1.0 × 10^−5^ in male), which were added to LG24 according to linkage analysis. Most of these markers (14/17) were located in the 0–11.863 cM sex QTL interval with high LOD value (LOD > 80), which suggest that the region may be a potential sex determining region (SD) (**Supplementary Figure S1** and **Supplementary Table S7**).

**FIGURE 2:**
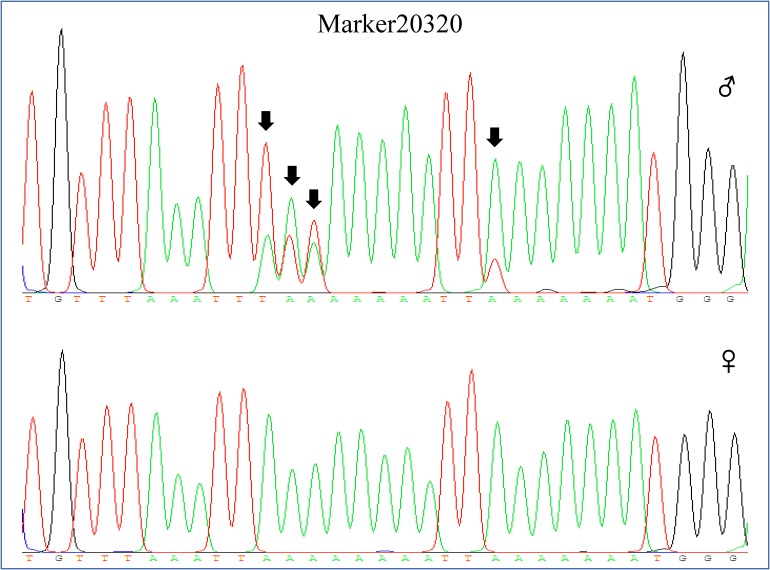
Genotype of Marker20320 in male and female individual by sequencing. The genotype of Marker20320 was heterozygous in male (160:A/T;161:A/T;162:A/T;170:A/T, sequencing chromatograms showed two peaks on the SNP site) and homozygous in female (160:A;161:A;162:A;170:A). 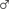 represents male individual and 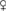 represents female individual. The black arrow indicates the SNP position.

**FIGURE 3:**
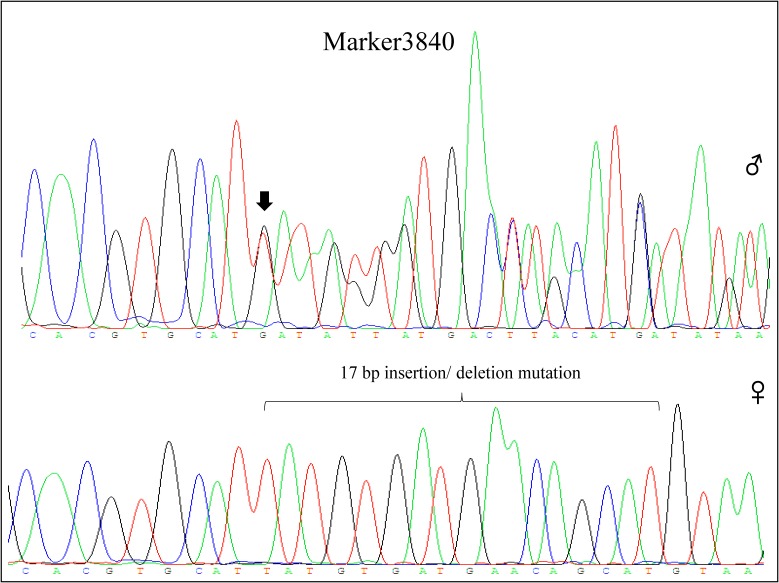
Genotype of Marker3840 in male and female individual by sequencing. Marker3840 included a 17 bp insertion/deletion mutation, which was heterozygous in male (65:T/-;66:A/-;67:T/-;68:G/-;69:T/-;70:G/-;71:A/-;72:T/-;73:G/-;74:A/-;75:A/-;76:C/-;77:A/-;78:G/-;79:C/-;80:A/-;81:T/-, sequencing chromatograms showed disorderly behind the site of insertion/ deletion mutation) and homozygous in female (65:T; 66:A; 67:T; 68:G; 69:T; 70:G; 71:A; 72:T; 73:G; 74:A; 75:A; 76:C; 77:A; 78:G; 79:C; 80:A; 81:T). 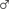 represents male individual and 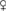 represents female individual. The black arrow indicates the insertion/deletion mutation position.

Among these markers, two markers were perfectly associated with sex (Marker20320 and Marker3840, lm x ll type), which were further validated in randomly selected 30 females and 30 males of different populations (two full sibling families and one wild population). The results showed that the two markers were always homozygous in females and always heterozygous in males at the family level (**Figures 2**, **3**). Even at the population level, the markers were still tightly associated with sex (*p* = 0, χ^2^ test), and almost all males are heterozygous and females are homozygous (>95%) (**Table 2**).

**Table 2:** Validation data of sex markers in different populations.

	1^#^ full sibling family		2^#^ full sibling family		The wild population	
	Female	Male	Female	Male	Female	Male
Marker20320						
Homozygosity	30	0	30	0	28	0
Heterozygosity	0	30	0	30	2	30
*Marker3840*						
Homozygosity	30	0	30	0	29	1
Heterozygosity	0	30	0	30	1	29

### qPCR Analysis of Sex-Related Genes

**FIGURE 4:**
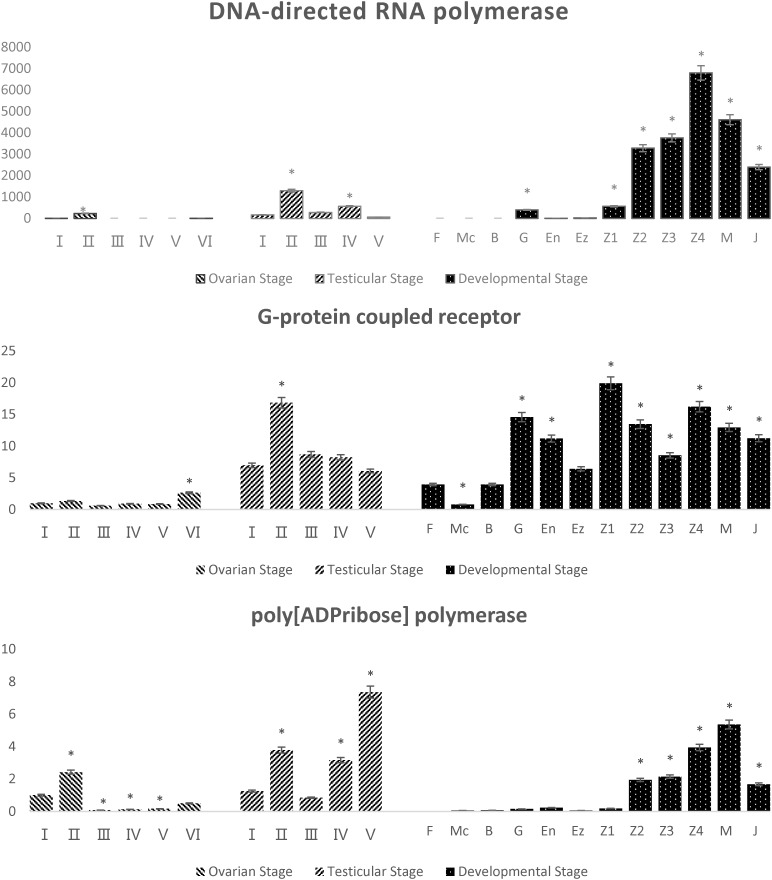
Quantitative real-time PCR (qPCR) analysis of potential sex-related genes. Testicular samples contain five stages (I–V) and ovarian samples contain six stages (I–VI). Twelve different developmental stages from fertilized eggs to young crabs including fertilized egg stage (F), multicellular stage (Mc), blastula stage (B), gastrulation stage (G), egg-nauplius stage (En), egg-zoea stage (Ez), zoea stage (Z1–Z4), megalopal stage (M), and juvenile crab stage (J). Asterisk (^∗^) marks the significant difference between each specific developmental period and the control group (*p* < 0.05).

Based on the marker sequence comparison and a method using candidate genes, three potential sex-related genes were found. They were DNA-directed RNA polymerase (match with Marker20320), G-protein coupled receptor (match with Marker3840) and poly[ADPribose] polymerase (match with Marker10494). All of the three genes were expressed higher in testis than ovary (**Figure 4**), the average expression of these genes in testis was 12.1, 7.6, and 4.6 times of that in ovary, respectively. During the different stages of embryonic development, DNA-directed RNA polymerase and poly[ADPribose] polymerase showed a similar pattern of expression, which began to upregulate expression at the Z2 stage (upregulate 8.3 and 34.6 times compared to the first sample that can be detected during different developmental stages, respectively) and reach a peak at the Z4 and C stages (17.1 and 70.2 times, respectively), then the expression decreased in the J stage (6.0 and 29.8 times, respectively). The expression of the G-protein coupled receptor appeared as an irregular wave pattern, and reached a peak in expression at Z1 stage (**Figure 4**).

## Discussion

*Portunus trituberculatus* is an important marine cultured crustacean species. As female crabs are of a higher economic value, clarifying the mechanism of sex determination has great implications both in theory and practice, and has attracted a lot of attention from biologists. In the present study, association analyses and SD analysis were first applied to identify QTLs for sex and the corresponding sex-associated markers based on a high-density genetic linkage map. The finding in this study will be useful for revealing the sex chromosome characteristics in future studies.

With the combination of next-generation sequencing (NGS) and restriction digestion enzymes, RAD-Seq offers the possibility for generating 1000s of SNPs in a short time, and these could be used for sex QTL mapping and in sex-associated markers identification (Liu et al., 2018). Based on a previously constructed high-density genetic linkage map via SLAF-seq (Lv et al., 2017), sex QTL was found to be located on LG24 (0.87–44.591 cM) in this study. As far as we know, this is the first time that sex QTL has been specifically located on a single chromosome in the crab, which suggested that LG24 may be a potential sex chromosome. The female and male LG24 are similar in size, and the two sex chromosomes have a large number of homologous genes. No difference was found in the recombination rates (female:male = 0.97) (Lv et al., 2017). Combined with the previous karyotype studies in the crab (no sex chromosome was found) (Zhu et al., 2005), presumably the full sex-associated region occupies a very small region on the sex chromosome. However, the identification of accurate sex QTL regions requires further verification in multiple families.

Twenty markers in the sex QTL-region (9.431–44.591cM of the LG24) showed significant associations with sex via association analysis. Among which, 19 markers were male heterozygosity (genotyped as lmxll), and strongly suggested that the crab may belong to the male heterogametic XY sex determination system. We noticed that some nnxnp makers (such as Marker33022) with the highest LOD score were not associated with sex via association analysis. It is believed that the phenomenon was mainly due to the multiple-QTL MQM of MapQTL 4.0 software, which provided a sensitive approach for mapping QTL in experimental populations, and added higher statistical power compared to many other methods (Arends et al., 2010). The software may estimate that the QTL exists somewhere upstream or downstream of the lmxll markers (sex-related), within a location that happens to be occupied by nnxnp markers (sex unrelated). Interestingly, the result was completely opposite to a previous study in *E. sinensis*, an important ecological and economic species with a supposedly ZW sex determination system (Cui et al., 2015). In comparison with vertebrates, sex determination mechanisms in crustaceans are more diverse (Ford, 2008), so it is not surprising that the two crabs have different sex determination systems. With the help of genome sequencing, the two species will be ideal research materials for studying the XY and ZW sex determination system in crab.

Although 20 markers in the QTL region were significantly associated with sex, none of them showed complete association with sex determination. One of the main reasons was that the sex markers complete associated with sex could be considered as ‘bad’ markers to be excluded from genetic map construction. In this work, sex markers tended to be deviated from Mendelian segregation ratio due to the sex ratio of our mapping population was not 1:1 (the observed ratio of female to male was 2.31:1 caused mainly by the higher mortality rate of male offspring during the mating period), which were often excluded from linkage analysis before the construction of genetic linkage maps. Therefore, some sex markers might be omitted only through QTL mapping analysis based on the genetic linkage map. For more extensive and more accurate finding sex markers, we further screened the sex markers in addition to the markers on the genetic linkage map by SD analysis. Finally, two markers perfectly associated with sex were identified, which also showed absolute female homozygosity and male heterozygosity in the QTL mapping population, further support for the conclusion of an XY sex determination system in the crab.

In the previous study, the recombination rate was reported to be high in crustacea (Zhang et al., 2007); the detected sex-associated markers may cross over frequently during meiosis. Consequently, the sex-associated markers identified in one family may disappear in another family (Qiu et al., 2017). Nevertheless, Marker20320 and Marker3840 were detected fully associated with sex in both of the full sibling families, which were located at 4.348 cM in LG24b (**Supplementary Figure S1**), the most significant region associated with sex (*PVE* = 100). Even at the population level, the markers were still tightly associated with sex (*p* = 0, χ^2^ test). To our knowledge, these two markers are believed to be most closely linked to sex in *P. trituberculatus*.

Three potential sex-related genes were identified based on bioinformatics and the candidate genes. A G-protein coupled receptor was found via Marker3840, which has previously been shown to be essential for male courtship (Li et al., 2011), probably by modulating the central nervous processing of sex pheromone through the action of one or both of its ligands (Abrieux et al., 2013). A DNA-directed RNA polymerase was found via Marker20320, which showed a high association with sex in turbot (*Scophthalmus maximus*) (Taboada et al., 2014). Poly[ADPribose] polymerase was found via Marker10494, which was an important constituent protein of SOX2 and associated with gonad development (Lai et al., 2012; Taboada et al., 2014). All of the three genes were expressed more in testis than ovary, suggesting that these genes were important in spermatogenesis or sex characteristics in males. It was worth noting that these genes began to up-regulate expression (DNA-directed RNA polymerase and Poly[ADPribose] polymerase) or expressed peak values (DNA-directed RNA polymerase and G-protein coupled receptor) in the zoea period, which suggested that the zoea period may be a critical period of sex differentiation.

## Conclusion

In this study, a highly significant QTL specifically located on LG24 (LOD > 14) was identified for the first time based on a high-density linkage map. Heterogametic genotypes of sex-associated markers in male support the XY sex determination mechanism. Two sex-specific markers at the family level were identified via SD analysis, which were known to be most closely linked to the sex of *P. trituberculatus*. Three sex-related genes with a potential function in spermatogenesis were identified. With the help of genome sequencing, our results will contribute to the fine-mapping of sex-determining genes and clarify sex determination mechanisms of *P. trituberculatus* in the future.

## Author Contributions

JLi and PL conceived and supervised the project. JLv and DS supplied the experimental animals. JLv contributed to the QTL mapping, association analysis, segregation distortion analysis, and wrote the manuscript. PH and LS contributed to the qPCR analysis. All authors read and approved the final manuscript.

## Conflict of Interest Statement

The authors declare that the research was conducted in the absence of any commercial or financial relationships that could be construed as a potential conflict of interest.

## Supplementary Material

FIGURE S1 LG24 of *P. trituberculatus.* LG24a represents the original linkage group 24 (LG24), and LG24b represents the new LG24 adding sex markers (including Marker3840 and Marker20320). Red fonts represent newly added sex markers. Green twill box represent potential sex determining region (SD).

TABLE S1 Primer used in this experiment.

TABLE S2 Quantitative trait locus (QTL) mapping of sex determination region in Portunus trituberculatus.

TABLE S3 Marker genotyping data via SLAF analysis.

TABLE S4 Segregation distortion markers in female group.

TABLE S5 Segregation distortion markers in male group.

TABLE S6 The makers showing segregation distortion in opposition directions between the sexs.

TABLE S7 The marker sequences on LG24.
